# Ethnopharmacological Potential of Phytochemicals and Phytogenic Products against Human RNA Viral Diseases as Preventive Therapeutics

**DOI:** 10.1155/2023/1977602

**Published:** 2023-02-20

**Authors:** Anamika Paul, Nilanjan Chakraborty, Anik Sarkar, Krishnendu Acharya, Anuj Ranjan, Abhishek Chauhan, Shilpi Srivastava, Akhilesh Kumar Singh, Ashutosh Kumar Rai, Iqra Mubeen, Ram Prasad

**Affiliations:** ^1^Department of Botany, Scottish Church College, Kolkata 700006, India; ^2^Molecular and Applied Mycology and Plant Pathology Laboratory, Department of Botany, University of Calcutta, Kolkata 700019, India; ^3^Academy of Biology and Biotechnology, Southern Federal University, Stachki 194/1, 344090 Rostov-on-Don, Russia; ^4^Amity Institute of Environment Toxicology and Safety Management, Amity University, Noida, U.P., India; ^5^Amity Institute of Biotechnology, Amity University Uttar Pradesh, Lucknow Campus, Lucknow, India; ^6^Department of Biotechnology, Mahatma Gandhi Central University, Motihari, 845401 Bihar, India; ^7^Department of Biochemistry, College of Medicine, Imam Abdulrahman Bin Faisal University, Dammam, Saudi Arabia; ^8^State Key Laboratory of Rice Biology, and Ministry of Agriculture Key Laboratory of Molecular Biology of Crop Pathogens and Insects, Institute of Biotechnology, Zhejiang University, Hangzhou 310058, China; ^9^Department of Botany, Mahatma Gandhi Central University, Motihari, 845401 Bihar, India

## Abstract

RNA viruses have been the most destructive due to their transmissibility and lack of control measures. Developments of vaccines for RNA viruses are very tough or almost impossible as viruses are highly mutable. For the last few decades, most of the epidemic and pandemic viral diseases have wreaked huge devastation with innumerable fatalities. To combat this threat to mankind, plant-derived novel antiviral products may contribute as reliable alternatives. They are assumed to be nontoxic, less hazardous, and safe compounds that have been in uses in the beginning of human civilization. In this growing COVID-19 pandemic, the present review amalgamates and depicts the role of various plant products in curing viral diseases in humans.

## 1. Introduction

The continuous increase in the human population has made globalization and contact between people, domestic animals, and wildlife inevitable. This increased connectivity between man and the wild has led to some devastating diseases from wildlife reservoirs with a heavy mortality toll. Some of them like the human immunodeficiency virus (HIV), H1N1 influenza, the highly pathogenic H5N1 avian influenza, Nipah virus, Hendra virus, severe acute respiratory syndrome coronavirus (SARS-CoV), Ebola virus (EBOV), and more recently, severe acute respiratory syndrome coronavirus 2 (SARS-CoV-2) have created havoc in the world [1, 2]. Studies of decades show that the RNA viruses are the most common class of virus, which are often highlighted as the preeminent member of pathogen class that is behind new human diseases, with a rate of 2 to 3 novel viruses being exposed each year [3, 4]. Meanwhile, vaccine development is introduced each time after the advent of new threats. Currently, a number of vaccines are considered to be a critical component in the prevention of viral infections [5], but most of them show side effects, and many of the viruses acquired resistance against them [6]. Therefore, the unavailability of potent vaccines increased mortality numbers. Vaccines are approximately only 50% effective; hence, there is a strong need for antiviral compounds with the ability to suppress viruses, devoid of any or any major side effects [6]. Fascinatingly, traditional medicine has been used in several parts of the worlds like in Asian, African, European, and South American countries. Because the traditional healthcare system is easily culturally acceptable and relatively cheaper compared to costly orthodox medicines [7]. Starting from past to present, wild plants are used traditionally due to having their medicinal properties in between rural communities and the societies to making a bridge in generation after generation, standing in this present scenario where the communication technology developed faster and spread data in wide distances that helps the people to enrich their traditional knowledge, which are essential in our daily life [8]. In addition, as compared to the orthodox system, plant-based natural pharmacotherapy can be used easily as proper alternatives for treating viral diseases [9]. In this review article, our main motive is to highlight the protective measures of RNA viral diseases in humans by using easily available medicinal plants, crude extracts, or active compounds.

## 2. Potential Pharmacological Targets of RNA Viruses in Host

RNA viruses contain RNA molecule to carry their genetic information. However, it also contains the information required for the synthesis of its own protein. These proteins may help in replication and spread to other susceptible host. Due to their reduced coding capacity, they are dependent on host cell to complete multiplication cycle [10]. This generalized strategy is followed by many other RNA viruses like human immunodeficiency virus (HIV) [11]. Corona virus disease 2019 (COVID-19) caused by the RNA virus severe acute respiratory syndrome coronavirus 2 (SARS-CoV-2) is a serious threat to mankind as well as the world economy [2]. Most interestingly, host RNA-binding protein is attached with the specific architecture of RNA. In case of SARS-CoV-2, infection cycle is started by the interaction of receptor-binding domain (RBD) with angiotensin-converting enzyme 2 (ACE2) receptors present on the cell surface of human. After binding, transmembrane protease and serine 2 cleaved S protein facilitate the entry of virus. The SARS-CoV-2 particles enter the host through either direct membrane fusion or endocytosis. After its translation to polypeptides, the main protease (M^pro^) and papain-like protease (PL^pro^) cleaved the translated polypeptides to release nonstructural proteins (nsps), whereas RNA-dependent-RNA polymerase (RdRp) facilitates RNA transcription and replication. On the other hand, its mRNA is translated to produce structural and accessory proteins in endoplasmic reticulum (ER). Then, structural proteins are assembled with nucleocapsid into the secretory vesicles (lumen). Finally, the assembled SARS-CoV-2 particles were released by following the exocytosis method [12].

## 3. Efficiency of RNA Viruses for Pathogenesis

**Figure 1:**
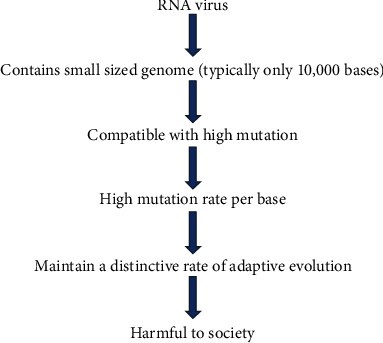
Natural outline of RNA viruses in causing disease.

**Figure 2:**
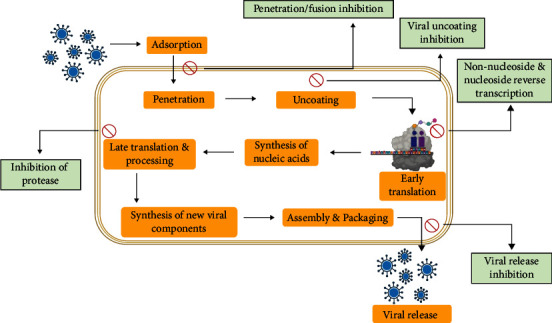
Basic mode of action and related inhibitory factors of virus.

Till now, 13 families of single-stranded RNA (ssRNA) and 1 family of double-stranded RNA (dsRNA) virus have been included in virus classification [13]. ssRNA viruses are further classified with having either positive or negative sense strands [14]. Those viruses are capable to modify cellular metabolism upon successful infection [15]. They invariably use host cell ribosomes for the production of protein and related enzymes [16]. Interestingly, RNA viruses have tremendous adaptability to a new environment. They can also be able to handle harsh situations related to different selection pressures encountered, though the selective pressures not only include the host's immune system and defense mechanisms but are also encountered by challenges created through the application of drugs such as protease inhibitors of hepatitis B and C virus and inhibitors of HIV-1 reverse-transcriptase [1]. Due to exceptionally shorter generation times and faster evolutionary rates, in a very short-time span, only the RNA viruses are able to transmit a disease to new host species [1]. The fast evolutionary rate of RNA viruses incurred due to the rapid rate of replication errors [17]. It is justified by many studies that the rate of mutation of RNA viruses is about six times greater than that of their cellular hosts. This is remarkably due to high mutation rate, which helps to maintain a distinctive rate of adaptive evolution that is shown in Figure 1 [1]. Basic mode of infection related to other factors are depicted in Figure 2.

## 4. Plant-Based Therapies against RNA Viral Disease

**Figure 3:**
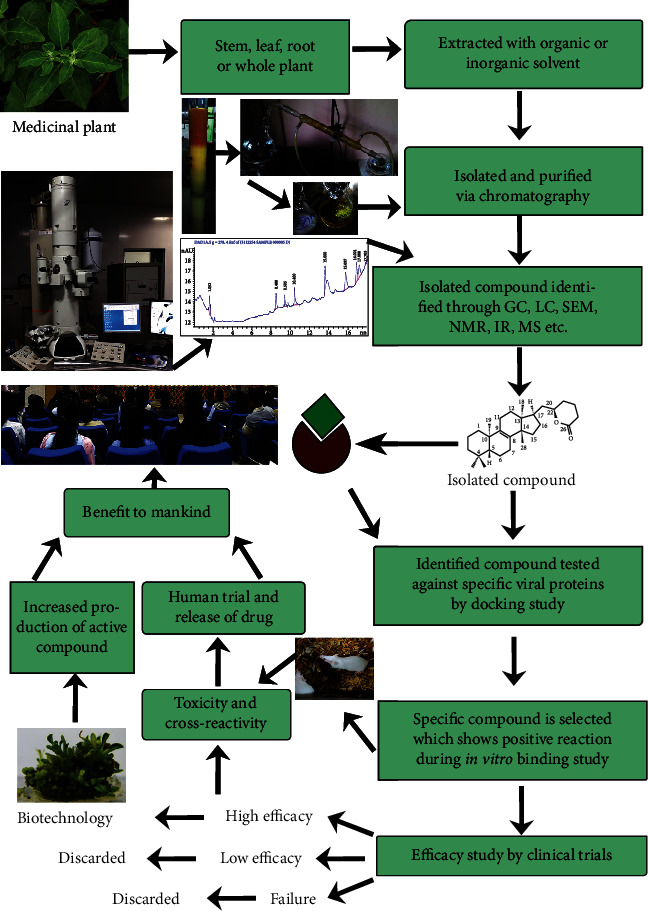
Comprehensive scheme of natural drug discovery from the medicinal plants.

Medicinal plant (MP) contains a substantial amount of pharmacologically important secondary metabolites that can be used for therapeutic purposes. Since the very past, different plant parts or extracts had been utilized by local people to prevent many diseases up till now [18]. Interestingly, viral infections with sharp mortality and morbidity rates are one of the most key concerns of human deaths worldwide [6]. In this irresistible situation of global pandemic, world research has been shifted to find out potential vaccines [4]. Due to the lack of vaccines and other standard therapies, viral diseases grow rapidly throughout the world. In this situation, discovery of the novel antiviral drugs is of utmost importance. Moreover, cost-effectiveness and high efficacy are always a matter of concern for drug discovery [19]. In many cases, the synergistic effect of plant extract in combinations with other compounds has shown better results against these diseases [20]. Since the very early times, active compounds were isolated and identified by following the bioassay-guided fractionation techniques that take longer time and most of the times do not show positive results. The recent methods of drug discovery are more efficient due to quick isolation and faster progression of novel drug formulation that also includes rapid clinical trials [21]. A concise strategy for natural drug discovery is shown in Figure 3.

On the other hand, commonly used natural plant products in Ayurveda are clearly mentioned in Susruta Samhita and Charaka Samhita or it can be obtained through traditional knowledges [22]. Conventionally used extracts of specific plant parts like roots, bark, stem, seeds, fruits and flowers, dietary supplements, plant derivatives (phyto-constituents), and nutraceuticals found wide applications in the treatment of wide range of diseases. Besides that, the survey report indicates that among commonly used medicines, one quarter of the compounds are isolated from plant sources [22]. Hence, the scientists working on drug discovery are trying to focus on the medicinal plants used in ethnobotany and trying to document them to establish their natural, inherent, and positive effects against specific diseases [7, 23]. But in comparison with the investigation of antimicrobial properties, the investigation of plant-derived antiviral substances is insufficient [24, 25].

### 4.1. Herbal Extracts against RNA Viral Diseases of the Human

Traditionally, medicinal plants are being used to cure various human diseases. Most of the Asian countries like China, Japan, and India have a great past history of usage of medicinal plant and mushroom extracts [26]. Amalgamation of conventional knowledge and advance research techniques in the field of natural drug discovery imports a huge number of novel products from plants. Due to less side effects and toxicity, it has been preferred by the common users. Not only that the cost-effectiveness of those natural products provides a value addition and also increased its accessibility [27]. Those products can be able to provide broad spectrum protective measures to numerous health hazards including viral infections. In case of the treatment of SARS-CoV-2, a formulation composed of 6.6% of each of the 15 plants, viz., *Zingiber officinale* (ginger), *Adhatoda vasica* (malabar nut), *Piper longum* (long pepper), *Andrographis paniculata* (bitterweed), *Tragia involucrata* (Indian stinging nettle), *Syzygium aromaticum* (clove), *Terminalia chebula* (chebulic myrobalan), *Hygrophila auriculata* (kokilaksha), *Cyperus rotundus* (java grass), *Plectranthus amboinicus* (Indian borage), *Clerodendrum serratum* (bharangi), *Tinospora cordifolia* (guduchi), *Saussurea costus* (costus), *Sida acuta* (teaweed), and *Anacyclus pyrethrum* (akarkara), has been recommended by the Ministry of AYUSH (Ayurveda, Unani, Yoga, and Naturopathy, Siddha, and Homoeopathy), Government of India [28]. The antiviral activity of many plant extracts and compounds of plant origin has been reported, which includes aqueous extracts of *Cassine xylocarpa* stem (Celastraceae), *Maytenus cuzcoina* root & bark (Celastraceae) [9], and crude methanolic extract of roots of four Lamiaceae members, viz., *Thymus carmanicus* (Thyme), *Thymus vulgaris*, *Thymus kotschyanus*, and *Thymus daenensis* [9, 29], which are used to cure HIV infection; aqueous extract of *Nerium oleander* (kaner, Apocynaceae) is used against Poliovirus type 1 [30]; methanolic extract of *Bryophyllum pinnatum* (life plant, Crassulaceae), *Mondiawhitei* (white's ginger, Periplocaceae), *Terminalia ivorensis* (black afara, Combretaceae), *Ageratum conyzoides* (goat weed, Asteraceae) are used to cure echovirus [7]; aqueous and ethanol extracts of dried leaves of *Andrographis paniculata* (Acanthaceae) and *Gynostemma pentaphyllum* (poor man's ginseng, Cucurbitaceae) are used to prevent avian influenza virus (H5N1) [31] etc.  Table 1 summarizes the use of plant extracts against the human RNA-viral diseases.

There is not a single medicine, which is globally accepted for the treatment of dengue fever. However, extracts obtained from different plants are expected to lower the severity of dengue viruses by increasing the platelet count, which includes *Carica papaya* leaf extract that is used by the local people of North Eastern plain zone of India, Madhya Pradesh [33, 56], whole plant of *Andrographis paniculata* (bitterweed), *Alternanthera sessilis* (sessile joyweed), *Achyranthus aspera* (prickly chaff flower), and *Solanum xanthocarpum* (yellow-fruit nightshade), and leaf and bark of *Calotropis procera* (calotrope) used in Bihar by traditional healers [32, 33]. In some clinical trials, it has been confirmed that the administration of leaf extract of *Carica papaya* (papaya) on patients with dengue fever increases their platelet count [33, 45]. More specifically, as we are facing SARS-CoV-2 in present days, so it is required to know the name of the phytochemicals and their classes to check the intensity of this virus [57].  Table 2 represents a list of phytochemicals acting against various targets of SARS-CoV-2, where most of the phytochemical binds with spike protein ACE2 (like anthraquinone, emodin, rhein, and cinnamaldehyde) or inhibits main protease (M^pro^) (like somniferine, isorientin, and gallocatechin-3-gallate) [57].

Ethanolic and methanolic extracts of *Ampelocissus tomentosa* (root), *Clerodendrum serratum* (whole plant), and *Terminalia chebula* (leaves) are effective against chikungunya virus, yellow fever virus, and entero virus, respectively [50]. Flavonoid extract of *Litchi chinensis* inhibits 3CL^pro^ [51, 68], and plant extracts of *Juniperus oxycedrus* (cade juniper), *Laurus nobilis* (bay tree), *Thuja orientalis* (thuja) act as viral growth inhibitor [51] against SARS-CoV. Ethanolic extract of *Broussnetia papyrifera* (paper mulberry) containing Kazinol F and Broussochalcone A, and 3′-(3-methylbut-2-enyl)-3′,4,7-trihydroxyflavane showed noncompetitive inhibition on papain like protease (PLpro) of MERS-CoV and SARS-CoV, respectively [69, 70]. The flavonoids like cinnamaldehyde, chrysin, and anethole bind with spike protein ACE2 of SARS-CoV-2 virus [59, 62]; tinocordiside, somniferine, and withanoside V [64] inhibit M^pro^ protease, which cleaved translated polypeptides to liberate nonstructural proteins.

### 4.2. Plant-Derived Active Compounds for Prevention of RNA Viral Diseases in Human

**Figure 4:**
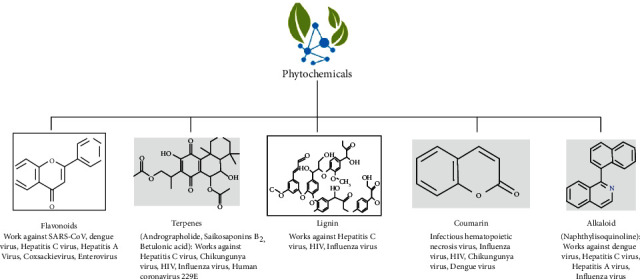
Important phytochemicals obtained from medicinal plants.

Nowadays, huge proportions of global population choose easily available natural product from their nearby sources to get relief from the emerging health problems. This awareness of common people has induced the interest of the scientists to invent new formulations of plant extracts on the basis of traditional ethnopharmacological knowledge. Sequentially, many pharmaceutical companies are now engaged to produce natural antimicrobial formulations to meet the demand of the global market [71]. Ignatov (2020) explained that high levels of potassium reported from *Moringa oleifera* helps the patient of COVID-19 to decrease the rate of infection caused by the SARS-CoV-2 virus. Different plants induce the production of numerous secondary metabolites such as phenolics, essential oils, glycosides, coumarins, alkaloids, terpenoids, and peptides mainly in response to microbial infections or to avoid herbivores and other organisms (Figure 4).

These metabolites have been accepted to play an essential role in boosting of the immune system and manifesting of antiviral potential [27, 72]. There are several phytochemicals, which are found to be effective against different RNA viruses also; some of which are listed in Table 3.

Few recurrent and non-recurrent viral infections include human immunodeficiency virus type 1 (HIV-1) and 2 (HIV-2) [27], hepatitis C virus [6], dengue virus [33], and poliovirus. Furthermore, the control and prevention of different emerging viral diseases are imposing a great challenge to the society [88]. About 500,000 plant species are estimated to be present in the world. Interestingly, 10-15% among them are being used as drugs and 10% as a source of food [89]. However, the rapid extinction of some species leads to irretrievable loss of potential phytochemicals and also imposes another serious threat. The anti-RNA viral activity of active compounds from some medicinal plants have been reviewed and summarized in Table 4.

Few preidentified compounds from different traditional medicinal plants of China showed protective actions against SARS-CoV-2 like betulinic acid, lignin, and sugiol on replication and chymotrypsin-like protease (3CLpro); coumaroyltyramine, cryptotanshinone, kaempferol, N-cis-feruloyltyramine, quercetin, and tanshinone IIa on papain like protease (PLpro) and 3CLpro; desmethoxyreserpine on replication, 3CLpro, and entry; dihydrotanshinone on entry and spike protein; and dihomo-c-linolenic and moupinamide on 3CLpro and PLpro, respectively, to prevent COVID-19 [70, 105]. Furthermore, theaflavin of *Camellia sinensis* (tea) shows biological action by binding on RNA-dependent RNA polymerase against SARS-CoV-2 [90]. Several drugs, which are previously approved by FDA for other diseases, are again used for the treatment of COVID-19 patients. These drugs include chloroquine and hydroxychloroquine, which are generally used as inhibitors of endosomal acidification fusion and auranoin for redox enzymes that helps to treat rheumatoid arthritis [106, 107]. Allyltrisulfide and allyldisulfide isolated from *Allium sativum* (garlic) act as ACE2 receptor inhibitors [51, 98]; herbacetin from *Linum usitatissimum* (flax) [51, 68], betulonic acid, savinin, and hinokinin from *Chamaecyparis obtusa* var. *formosana* (Taiwan yellow cypress) act as 3CL^pro^ inhibitor against SARS-CoV; *Scutellaria lateriflora* (blue skullcap) helps to prevent SARS-CoV by scutellarein as helicase inhibitor [51]. Furthermore, thymoquinone and andnigellimine from *Nigella sativa* (fennel flower) show the blocking of virus entry into pneumocyes and increasing the Zn^2+^ uptake to boost host immune response against SARS-CoV-2 [104]. The recent evidences showed that vicenin, isorientin, and 4′-O-glucoside 2^″^-O-p-hydroxybenzoagte obtained from *Ocimum sanctum* L. inhibit M^pro^ protease activity during SARS-CoV-2 viral infection [64].

In case of the treatment of Ebola virus, many alkaloids are isolated from *Atropa belladonna* that gives a positive result and cure of Ebola haemorrhagic fever (Ebola HF) [100, 101], whereas a sulfated polysaccharide, fucoidan isolated from *Cladosiphon okamuranus*, and sulphated polysaccharide (named kappa carrageenan) from whole plants of *Gymnogongrus griffithsiae* [94] worked against dengue virus type 2 (DENV-2). Cytarabine and matrine are two important active constituents found in the extract of Gillan's plants such as chuchaq and trshvash, respectively, whereas these compounds have the potentiality of better binding interaction with the receptors and show anti SARS-CoV-2 activity by inhibiting the initiation of viral infection [108].

There are three species of *Tephrosia* (viz. *T. crassifolia*, *T. madrensis*, and *T. viridiflora*), which can able to produce huge number of flavonoids and show antiviral activity against dengue virus. Among them, glabranine and 7-O-methyl-glabranine isolated from *T. madrensis* showed strong inhibitory effects on dengue virus replication in LLC-MK2 cells. Interestingly, moderate to low inhibitory effect was observed by methyl-hildgardtol A isolated from *T. crassifolia*. However, hildgardtol A of *T. crassifolia* and elongatine of *T. viridiflora* do not show any inhibitory effect on viral growth [94]. Honokiol (a lignin biphenol) derived from *Magnolia* tree has antiviral activity against serotype 2 dengue virus (DENV-2). This novel molecule can be able to interfere on the endocytosis of virus to reduce its double-strand RNA, by abrogating the colocalization of DENV envelope proteins. Inhibitory activity of honokiol was proved by suppressing the replication of DENV-2 in baby hamster kidney (BHK) and human hepatocarcinoma Huh7 cells [9, 47].

In the Chinese herbal medicine, extracts of *Lonicerae japonicae* (Japanese honeysuckle), *Flos* (ragged-robin), and *Fructus forsythia* (Lianqiao) are used together as an antimicrobial and anti-inflammatory agent. Interestingly, chito-oligosaccharide in combination with herbal mixture can also act as an anti-influenza agent [64].

## 5. Major Challenges and Future Perspectives

Nowadays, the acceptance of herbal medicines is increasing for many reasons as it is less expensive, with less or null side effects and better patient tolerance. From the past to recent studies, it can be speculated that quality and safety study of plant-derived compounds with medicinal importance is required. Therefore, scientific, accurate, and informative studies are obligatory for any potent compound, which have isolated from plants [21]. About half million plants with medicinal properties are estimated to be present around the world. Surprisingly as well as grievously, most of them are yet not investigated [109]. It is required to give more attention to taxonomic characters and identification of wide range of medicinal plants throughout the world [21]. Various pharmacologically important peptides or proteins obtained from different medicinal plants can be introduced in the production of vaccines and therapeutics. The main challenge in this field of research is the extraction procedure. In this connection after establishment of a compound as a potent therapeutic agent, researchers have to increase its production in both *in vitro* and *ex vitro* conditions. This is also very challenging but can be resolved by using sophisticated techniques of recombinant DNA technology, elicitation, etc. [26]. Along with these, identification of biosynthetic pathway of that particular product and application of transgenic approach can be used as feasible way to enhance the production of that particular product. Different scientific approaches can also be taken to identify the newer plant-derived compounds with potential pharmacological activities. This can be considered as an emerging field of scientific research with global profit. Furthermore, it was already expected by [21] that within the year of 2020, the whole market in Asia-Pacific for herbal supplements reached more than US$115 billion and about more than that much turnover happened in 2021, i.e., US$140 billion [21]. It is also explained in the report of Asia Pacific Nutritional Supplements Market Size, Share & Trends Analysis Report with a report ID of GVR-4-68038-105-4 that it is expected that it will expand about 6.1% at a compound annual growth rate (CAGR) starting from 2022 to 2030. So, it is clear that the use of herbal supplements and ethnic medicines are increasing day by day. In the recent days, several RNA interference (RNAi)-based technologies and clustered regularly interspaced short palindromic repeats (CRISPR)-Cas technology have appeared to control viruses. It helps by either directly targeting the viral RNA or DNA or by inactivating plant host susceptibility gene [110]. The CRISPR-Cas system is a technology, which uses in diagnosis of various infectious RNA viruses like SARS-CoV-2 and HIV. This is undertaken as a potential technology for its accuracy both in therapeutic and molecular diagnosis [111].

## 6. Conclusion

Standing in the current circumstance of global pandemic of COVID-19, that causes huge damage of human health and also destroys economic backbone of a country, researchers are constantly trying to find out the way by which mankind will be safe from the fourth or fifth wave of virus onslaught. At this time of high crisis period, we all want something to eradicate or bypass the disease. We reviewed the plant extracts and/or extracted and identified active compounds from the medicinal plants that are useful for prevention of many RNA viral diseases, focusing on plant extracts and phytochemicals that have already reached in clinical trials and having highlighting properties to prevent the viruses. Along with disruption of global health, RNA viruses have immense potential to stop the economic growth of a country. However, for prevention of those diseases and to secure human populations, antiviral drug discovery is needed. Till date, many traditional medicinal plants were reported to have strong antiviral activities. Medicinal plants contain active compounds like alkaloids, saponins, flavonoids, tannins, polysaccharides, proteins, and peptides. These compounds can help in the prevention of viral diseases by blocking the virus entry and/or via the inhibition of viral replication at the different stages by regulating the enzyme actions as well and specially without having any known side effects. Finally, the improvement of newly discovered medicinal plant products is vital and required for controlling the constant threats of deadly contagious RNA viruses. This review enlightens some way with lesser hazard to combat in this epidemic era of RNA virus like COVID-19. It is necessary to further examine by highlighting drug delivery system of antiviral phytochemical to reach successfully in their intended site of action.

## Figures and Tables

**Table 1:** Plant extract used as anti-RNA viral activity in human.

S. no.	Family	Plant name	Antiviral activity against	Traditional use of plant	Name of city and countries where these were used	Extraction method and parts used	Mechanism of action/reduction of disease intensity
1.	Acanthaceae	*Andrographis paniculata* (Burm. f.) Nees [Bitterweed]	Dengue virus (DENV)	By tribal healers, ojha, baidya, medicine men of Bihar	Bihar (state of India) [32]	Crude extract of whole plant	Inhibits the activity of DENV-1 in infected Vero cells in *in vitro* assay [33]
2.	Acanthaceae	*Andrographis paniculata* (Burm. f.) Nees [Bitterweed]	Avian influenza virus (H5N1)	Aerial parts and roots used as traditional medicine in India, China, Thailand, and other Southeast Asian countries to treat many diseases	India, China, and Thailand (countries of Asia) [34]	Water and ethanol extract of leaves [31]	Induce upregulation of *IFN-β* and *TNF-α* mRNA expression that inhibit viral replication in Madin-Darby canine kidney (MDCK) cells in an *in vitro* investigation [31]
3.	Moraceae	*Ficus fistulosa* Reinw. ex BI. [Kelampung Bukit]	Hepatitis C virus	In folk medicine against respiratory disorder, convulsion, and tuberculosis [35]	80% population of some Asian and African countries and Bangladesh (country in South Asia) [36]	Ethanolic extract of leaves [37]	Inhibition of viral entry [37]
4.	Anacardiaceae	*Schinus molle* L. [American pepper]	Human immunodeficiency virus (HIV)	In folk medicine for treating ulcers, wounds, diarrhea, toothache, menstrual disorders, rheumatism, and respiratory problem that are found in many Brazilian medical literature, as stimulant, antitumor, antifungal, antiseptic, tonic, diuretic, anti-plasmodic, antioxidant, antibacterial agent	Brazil (country of South America) [38]	Crude methanolic extract of leaves [39]	Protects MT-2 T-lymphoblastoid cell from cytopathic effect of HIV [39]
5.	Cucurbitaceae	*Gynostemmapentaphyllum* (Thunb.) Makino [Southern Ginseng or Miracle Plant]	Avian influenza virus (H5N1)	In Southeast Asian countries as herbal medicine to treat diabetes, as antioxidant, antitumor, cholesterol-lowering agent, treatment of chronic tracheitis, bronchitis, infectious hepatitis, pyelitis and gastroenteritis in Chinese medicine	China, (countries in East-Asia) and southeast Asian countries [31]	Water and ethanol extract of dried leaves [31]	Induce upregulation of *IFN-β* and *TNF-α* mRNA expression that inhibit viral replication in Madin-Darby canine kidney (MDCK) cells in an *in vitro* investigation [31]
6.	Equisetaceae	*Equisetum giganteum* L. [Southern giant horsetail]	Human immunodeficiency virus (HIV)	In herbal medicine in Central and South America, diuretic agent in ethnomed [40] icine	Brazil (country of South America) and some other countries of Central and South America [41]	Crude methanolic extract of stem [39]	Protects MT-2 T-lymphoblastoid cell from cytopathic effect of HIV [39, 41]
7.	Zingiberaceae	*Curcuma longa* L. [turmeric]	Avian influenza virus (H5N1)	Traditional medicine in India, Bangladesh, Pakistan, and China, as antiseptic for cuts, bruises, and burns in South Asia	India, Bangladesh, and Pakistan (countries in South-Asia) and China (country in East-Asia) [42]	Water and ethanol extract of root [31]	Induce upregulation of *IFN-β* and *TNF-α* mRNA expression that inhibits viral replication in Madin-Darby canine kidney (MDCK) cells in an *in vitro* investigation [31]
8.	Apocynaceae	*Calotropis procera* (Aiton) W.T.Aiton [Calotrope]	Dengue virus (DENV)	By tribal healers, ojha, baidya, medicine men of Bihar	Bihar (state of India) [33]	Crude extract of leaf and bark [33]	Kills larvae of *Aedes aegypti* [43]
9.	Caricaceae	*Carica papaya* L. [papaya]	Dengue virus (DENV)	By traditional healers and local people of Uttar Pradesh, Madhya Pradesh; local practitioners and traditional healers of Goa, local people of north eastern plain zone of India	Uttar Pradesh, Madhya Pradesh, Goa (states of India) and north eastern plain zone of India (country in South Asia) [33, 44]	Leaf extract [45]	Increases platelets due to administration of extract [45]
10.	Meliaceae	*Azadirachta indica* A. Juss. [neem]	Dengue virus type 2 (DENV-2)	By traditional healers and tribals of various districts of Bihar	Bihar (state of India) [33]	Leaf extract [33]	*In vitro* and *in vivo* study showed reduction of virus [33]
11.	Zingiberaceae	*Kaempferia parviflora* Wall. ex Baker [Thai ginseng]	Avian influenza virus (H5N1)	In Thai medicine to treat leucorrhoea, oral disease, stomach discomfort, health promotion, as antifungal, antiflatulent, antiplasmodial agent, powder with ethanol used to cure peptic ulcers, diabetes, asthma	Thailand (country in Asia)	Water and ethanol extract of root [31]	Induce upregulation of *IFN-β* and *TNF-α* mRNA expression that inhibit viral replication in Madin-Darby canine kidney (MDCK) cells in an *in vitro* investigation [31]
12.	Magnoliaceae	*Magnolia officinalis* Rehder & Wilson [Houpo magnolia]	Dengue virus type 2	In eastern medicine, Chinese medicine Hou-Pu that have been used in analgesic, distension, or anxiety relief [46]	China (country in East Asia)	Methanolic extract of bark [47]	Inhibits intracellular DENV-2 replicon [47]
13.	Lamiaceae	*Thymus carmanicus* Jalas [Avishan]	Human immunodeficiency virus type 1 (HIV-1)	Decoction and infusion used for cold in Iranian traditional medicine, as anti-inflammatory, digestive, carminative agents	Iran (country in Western Asia)	Methanolic extract of root [29]	Effect on peripheral blood mononuclear cells (PBMCs) toxicity and HIV-1 replication [29]
14.	Lamiaceae	*Thymus vulgaris* L. [thyme]	Human immunodeficiency virus type 1 (HIV-1)	Decoction and infusion used for cold in Iranian traditional medicine, in folk medicine for asthma and bronchitis, as anti-inflammatory, digestive, carminative, antiseptic, antifungal, antiviral, antimicrobial agents	Iran (countries in Western Asia)	Methanolic extract of root [29]	Effect on peripheral blood mononuclear cells (PBMCs) toxicity and HIV-1 replication [29]
15.	Lamiaceae	*Thymus kotschyanus* Boiss. & Hohen. [thyme]	Human immunodeficiency virus type 1 (HIV-1)	Decoction and infusion used for cold in Iranian traditional medicine, as anti-inflammatory, digestive, carminative agents	Iran (country in Western Asia)	Methanolic extract of root [29]	Effect on peripheral blood mononuclear cells (PBMCs) toxicity and HIV-1 replication [29]
16.	Lamiaceae	*Thymus daenensis* Celak [thyme]	Human immunodeficiency virus type 1 (HIV-1)	Decoction and infusion used for cold in Iranian traditional medicine, as anti-inflammatory, digestive, carminative agents	Iran (countries in Western Asia)	Methanolic extract of root [29]	Effect on peripheral blood mononuclear cells (PBMCs) toxicity and HIV-1 replication [29]
17.	Myrtaceae	*Psidium guajava* L. [guava]	Avian influenza virus (H5N1)	In South-Eastern Nigeria to treat cough, malaria, stomach disorders, and loss of appetite	Nigeria (country in West Africa) [48]	Water and ethanolic extract of dried leaves [31]	Induce upregulation of *IFN-β* and *TNF-α* mRNA expression that inhibit viral replication in Madin-Darby canine kidney (MDCK) cells in an *in vitro* investigation [31]
18.	Verbenaceae	*Clerodendrum serratum* (L.) Moon. [Bharangi]	Yellow fever virus	Used in Yunani, Ayurveda, traditional Chinese medicine, Japanese Kampo medicine	China, Japan (countries in East Asia) [49]	Ethanolic and methanolic extract of whole plant [50]	Prevent viral infection through the bite of mosquito vector [50]
19.	Combretaceae	*Terminalia chebula* Retz. [Chebulic myrobalan]	Enterovirus	Used in Yunani, Ayurveda, traditional Chinese medicine, Japanese Kampo medicine	China, Japan (countries in East Asia) [49]	Ethanolic and methanolic extract of leaves [50]	Inhibits viral replication [50]
20.	Vitaceae	*Ampelocissus tomentosa* (Heyne ex Roth) Planch. [hairy wild grape]	Chikungunya virus	Used in Yunani, Ayurveda, traditional Chinese medicine, Japanese Kampo medicine	China, Japan (countries in East Asia) [49]	Ethanolic and methanolic extract of root [50]	Inhibits viral replication [50]
21.	Crassulaceae	*Kalanchoe pinnata* (Lam.) Pers. [flaming Katy]	Chikungunya virus	Used in Yunani, Ayurveda, traditional Chinese medicine, Japanese Kampo medicine	China, Japan (countries in East Asia) [49]	Ethanolic and methanolic extract of leaves [50]	Inhibits viral replication [50]
22.	Cupressaceae	*Thuja orientalis* L. [Chinese thuja]	Severe acute respiratory syndrome coronavirus (SARS-CoV)	In traditional medicine and homeopath, to treat bronchitis, skin infection, excessive menstruation, arthritic pains, coughs, dysentery, named as Chinese Thuja, in other regions of the Asian continent	China (country in East Asia) and in other regions of the Asian continent	Oil extract of plant [51]	Inhibits viral replication [51]
23.	Cupressaceae	*Juniperus oxycedrus* L. [Cade juniper]	Severe acute respiratory syndrome coronavirus (SARS-CoV)	In infectious disease, colds, fungal infections, cough, gynecological disease, wounds in Turkish folk medicine, as anti-inflammatory and antinociceptive	Turki (lying partly in Asia and partly in Europe) [52]	Oil extract of plant [51]	Inhibits viral replication [51]
24.	Elaeagnaceae	*Hippophae rhamnoides*L. [Sea buckthorn]	Dengue virus type-2 (DENV-2)	In Tibetan traditional and Chinese medicine	Tibet is a part of China (country of East Asia) [53]	Alcoholic extract of leaves [33]	Decreases THF-*α*, increases INF-*γ* production, increases cell viability in *in vitro* assay against dengue virus type-2 [33]
25	Menispermaceae	*Tinospora cordifolia* (Thunb.) Miers [Guduchi]	Dengue virus (DENV)	In traditional folk medicine of India for treating diabetes	India (country in South Asia) [54]	Decoction of stems [55]	Reduce inflammation and fever, enhance the killing ability of macrophages [55]

**Table 2:** List of phytochemicals acting against various targets of SARS-CoV-2.

Sl no.	Phytochemical	Class of phytochemical	Targets	Reference
1.	Fisetin	Flavonoid	Bind with spike protein like hACE2-S	[58]
2.	Quercetin	Flavonoid	Bind with spike protein	[58]
3.	Dithymoquinone	Terpene	Inhibits spike glycoprotein-ACE2 interface	[59]
4.	Glycyrrhizic acid	Saponin	Binds with spike protein RBD-ACE2	[60, 61]
5.	Hesperidin	Flavonoid	Bind with spike protein ACE2	[62]
6.	Emodin	Flavonoid	Bind with spike protein ACE2	[62]
7.	Carvacrol	Phenol	Interacts with spike protein ACE2	[59]
8.	Rhein	Flavonoid	Bind with spike protein ACE2	[62]
9.	Kaempferol	Flavonoid	Bind with spike protein hACE2-S	[58]
10.	Cinnamaldehyde	Flavonoid	Interacts with spike protein ACE2	[59]
11.	Cinnamyl acetate	Styrene	Interacts with spike protein ACE2	[59]
12.	Chrysin	Flavonoid	Bind with spike protein ACE2	[62]
13.	Anthraquinone	Flavonoid	Bind with spike protein ACE2	[62]
14.	Anethole	Flavonoid	Interacts with spike protein ACE2	[59]
15.	Geraniol	Terpene	Interacts with spike protein ACE2	[59]
16.	L-4-terpineol	Terpene	Interacts with spike protein ACE2	[59]
17.	Andrographolide	Terpenoid	Inhibits M^pro^ protease	[63]
18.	Withanoside V		Inhibits M^pro^ protease	[64]
19.	Somniferine		Inhibits M^pro^ protease	[64]
20.	Epigallocatechin gallate	Phenol	Inhibits M^pro^ protease	[65]
21.	Gallocatechin-3-gallate	Phenol	Inhibits M^pro^ protease	[65]
22.	Tinocordiside		Inhibits M^pro^ protease	[64]
23.	Vicenin		Inhibits M^pro^ protease	[64]
24.	Isorientin		Inhibits M^pro^ protease	[64]
25.	Berbamine		Targets on spike protein ACE2	[66]
26.	Castanospermine		Reduced viral RNA	[67]
27.	Epicatechin gallate	Phenol	Inhibits M^pro^ protease	[65]

**Table 3:** Lists of phytochemicals against RNA viruses causing human diseases.

RNA type	Name of viruses	Family	Therapeutic phytochemicals	Reference
Positive-sense ssRNA	Dengue virus	*Flaviviridae*	Flavonoid, alkaloid, phenol, and coumarins	[73, 74]
	Hepatitis C virus	*Flaviviridae*	Flavonoid, alkaloid, lignan, terpenes, and terpenoids	[75, 76]
	Japanese encephalitis virus	*Flaviviridae*	Flavonoid	[77]
	Zika virus	*Flaviviridae*	Flavonoid	[78]
	Severe acute respiratory syndrome coronavirus (SARS-CoV)	*Coronaviridae*	Flavonoid (luteolin, curcumin, myricetin, quercetin), anthraquinone (emodin)	[51]
	Chikungunya virus	*Togaviridae*	Flavonoid, terpenes, coumarins, and terpenoids	[73, 76, 79]
	Coxsackievirus	*Picornaviridae*	Flavonoids	[80]
	Hepatitis A virus	*Picornaviridae*	Flavonoids and alkaloids	[81]
	Enterovirus	*Picornaviridae*	Flavonoids	[74]
	Human immunodeficiency virus	*Retroviridae*	Terpenes and terpenoid lignan, and coumarin	[82, 83]

Negative-sense ssRNA	Respiratory syncytial virus	*Paramyxoviridae*	Lignan	[84]
	Infectious hematopoietic necrosis virus	*Rhabdoviridae*	Coumarin	[85]
	Influenza virus (H3N2, H5N2, H5N1, H1N1)	*Orthomyxoviridae*	Flavonoid, alkaloid, lignan, coumarin, terpenes, and terpenoid	[86, 87]

**Table 4:** Medicinally important plant-derived active compounds used for curing human RNA viral diseases.

S. no.	Family	Plant name	Active compound	Family of virus	Virus name	Type of viral RNA	Human diseases	Reference
5.	Theaceae	*Camellia sinensis* (L.) Kuntze [tea]	Theaflavin	*Coronaviridae*	Severe acute respiratory syndrome coronavirus 2 (SARS-CoV-2)	Positive-sense ssRNA	Coronavirus disease 2019 (COVID-19)	[90]
6.	Acanthaceae	*Andrographis paniculata* (Burm. f.) Nees [Kalmegh]	Andrographolide	*Togaviridae*	Chikungunya virus (CHIKV)	Positive-sense ssRNA	Chikungunya fever	[91]
7.	Ancistrocladaceae	*Ancistrocladuskorupensis* D.W. Thomas & Gereau	Naphthylisoquinoline alkaloids from root bark	*Retroviridae*	Human immunodeficiency virus (HIV)	Positive-sense ssRNA	AIDS	[27]
8.	Berberidaceae	*Berbaris amurensis* Rupr. [Amur barberry]	Berbamine	*Coronaviridae*	Respiratory syndrome coronavirus-2 (SARS-CoV-2)	Positive-sense ssRNA	COVID-19	[66]
9.	Ancistrocladaceae	*Ancistrocladus congolensis* J, Léonard	Michellamine-type dimeric naphthylisoquinoline alkaloids from root bark	*Retroviridae*	Human immunodeficiency virus (HIV)	Positive-sense ssRNA	AIDS	[92]
10.	Fabaceae	*Castanospermum australe* A.Cunn & C.Fraser ex Hook. [Blackbean]	Castanospermine	*Coronaviridae*	Respiratory syndrome coronavirus-2 (SARS-CoV-2)	Positive-sense ssRNA	COVID-19	[67]
11.	Halymeniaceae	*Cryptonemiacrenulata* (J.Agardh) J.Agardh	Galactan	*Flaviviridae*	Dengue virus type 2, 3 and 4 (DENV-2, DENV-3, DENV-4)	Positive-sense ssRNA	Dengue (Breakbone fever)	[39]
12.	Apiaceae	*Bupleurum* sp.	Triterpene glycosides (named Saikosaponins B_2_)	*Coronaviridae*	Human coronavirus 229E (HCoV-22E9)	Positive-sense ssRNA	Nosocomial respiratory viral infection (NRVI)	[93]
13.	Fabaceae	*Tephrosia madrensis* Seem. [Hoarypea]	Glabranine and 7-O-methyl-glabranine from leaves and flowers	*Flaviviridae*	Dengue virus (DENV)	Positive-sense ssRNA	Dengue (Breakbone fever)	[94]
14.	Apiaceae	*Heteromorpha* sp.	Triterpene glycosides (named Saikosaponins B_2_)	*Coronaviridae*	Human coronavirus 229E (HCoV-22E9)	Positive-sense ssRNA	Nosocomial respiratory viral infection (NRVI)	[93]
15.	Moraceae	*Broussnetiapapyrifera* (L.) Vent. [paper mulberry]	Kazinol F, Broussochalcone A from roots	*Coronaviridae*	Middle Eastern respiratory syndrome coronavirus (MERS-CoV)	Positive-sense ssRNA	Middle Eastern respiratory syndrome	[69, 70]
16.	Nelumbonaceae	*Nelumbo nucifera* Gaertn. [lotus]	Isoliensinine	*Coronaviridae*	Respiratory syndrome coronavirus-2 (SARS-CoV-2)	Positive-sense ssRNA	COVID-19	[95]
17.	Nelumbonaceae	*Nelumbo nucifera* Gaertn. [lotus]	Liensinine	*Coronaviridae*	Respiratory syndrome coronavirus-2 (SARS-CoV-2)	Positive-sense ssRNA	COVID-19	[95]
18.	Moraceae	*Broussnetiapapyrifera* (L.) Vent. [paper mulberry]	3′-(3-Methylbut-2-enyl)-3′,4,7-trihydroxyflavane from roots	*Coronaviridae*	Severe acute respiratory syndrome coronavirus (SARS-CoV)	Positive-sense ssRNA	Severe acute respiratory syndrome (SARS)	[70]
19.	Polygonaceae	*Reynoutria multiflora* (Thunb.) Moldenke	Emodin	*Coronaviridae*	Severe acute respiratory syndrome coronavirus (SARS-CoV)	Positive-sense ssRNA	Severe acute respiratory syndrome (SARS)	[51]
20.	Ranunculacea	*Thalictrum podocarpum* Kunth ex DC. [*Meadow-rue*]	Hernandezine	*Coronaviridae*	Respiratory syndrome coronavirus-2 (SARS-CoV-2)	Positive-sense ssRNA	COVID-19	[96]
21.	Polygonaceae	*Rheum officinale* L. [Chinese rhubarb]	Emodin	*Coronaviridae*	Severe acute respiratory syndrome coronavirus (SARS-CoV)	Positive-sense ssRNA	Severe acute respiratory syndrome (SARS)	[51]
22.	Zingiberaceae	*Curcuma longa* L. [turmeric]	Curcumin	*Coronaviridae*	Severe acute respiratory syndrome coronavirus (SARS-CoV)	Positive-sense ssRNA	Severe acute respiratory syndrome (SARS)	[51]
23.	Nelumbonaceae	*Nelumbo nucifera* Gaertn. [lotus]	Neferine	*Coronaviridae*	Respiratory syndrome coronavirus-2 (SARS-CoV-2)	Positive-sense ssRNA	COVID-19	[95, 96]
24.	Myricaceae	*Myrica faya* Ait. [fire tree]	Myricetin	*Coronaviridae*	Severe acute respiratory syndrome coronavirus (SARS-CoV)	Positive-sense ssRNA	Severe acute respiratory syndrome (SARS)	[51]
25.	Meliaceae	*Toona sinensis* (A.Juss.) M.Roem. [Chinese mahogany]	Quercetin	*Coronaviridae*	Severe acute respiratory syndrome coronavirus (SARS-CoV)	Positive-sense ssRNA	Severe acute respiratory syndrome (SARS)	[51]
26.	Rubiaceae	*Cinchona officinalis* L. [Lojabark]	Quinacrine	*Coronaviridae*	Respiratory syndrome coronavirus-2 (SARS-CoV-2)	Positive-sense ssRNA	COVID-19	[97]
27.	Linaceae	*Linum usitatissimum* L. [Flax]	Herbacetin	*Coronaviridae*	Severe acute respiratory syndrome coronavirus (SARS-CoV)	Positive-sense ssRNA	Severe acute respiratory syndrome (SARS)	[51, 68]
28.	Lamiaceae	*Scutellaria lateriflora* L. [blue skullcap]	Scutellarein	*Coronaviridae*	Severe acute respiratory syndrome coronavirus (SARS-CoV)	Positive-sense ssRNA	Severe acute respiratory syndrome (SARS)	[51]
29.	Fabaceae	*Pterocarpus santalinus* L. f. [red sanders]	Savinin	*Coronaviridae*	Severe acute respiratory syndrome coronavirus (SARS-CoV)	Positive-sense ssRNA	Severe acute respiratory syndrome (SARS)	[51]
30.	Cupressaceae	*Chamaecyparis obtusa* var. *formosana* [Taiwan yellow cypress]	Betulonic acid, Savinin, Hinokinin	*Coronaviridae*	Severe acute respiratory syndrome coronavirus (SARS-CoV)	Positive-sense ssRNA	Severe acute respiratory syndrome (SARS)	[51]
31.	Celastraceae	*Triterygium regelii* Sprag. & Takeda [Regel's threewingnut]	Iguesterin, Tingenone, Pristimerin, Celastrol	*Coronaviridae*	Severe acute respiratory syndrome coronavirus (SARS-CoV)	Positive-sense ssRNA	Severe acute respiratory syndrome (SARS)	[51]
32.	Brassicaceae	*Isatisindigotica* fortune [Chinese woad]	Sinigrin, Hesperetin	*Coronaviridae*	Severe acute respiratory syndrome coronavirus (SARS-CoV)	Positive-sense ssRNA	Severe acute respiratory syndrome (SARS)	[51]
33.	Amaryllidaceae	*Allium sativum* L. [garlic]	Allyl trisulfide, allyl disulfide	*Coronaviridae*	Severe acute respiratory syndrome coronavirus (SARS-CoV)	Positive-sense ssRNA	Severe acute respiratory syndrome (SARS)	[51, 98]
34.	Acanthaceae	*Andrographis paniculata* (Burm. f.) Nees [Kalmegh]	Andrographolide	*Coronaviridae*	Respiratory syndrome coronavirus-2 (SARS-CoV-2)	Positive-sense ssRNA	COVID-19	[88]
35.	Meliaceae	*Aglaia* sp.	Silvestrol	*Coronaviridae*	Human coronavirus 229E (HCoV-229E)	Positive-sense ssRNA	Nosocomial respiratory viral infection (NRVI)	[70, 99]
36.	Meliaceae	*Aglaia* sp.	Silvestrol	*Coronaviridae*	Middle Eastern respiratory syndrome coronavirus (MERS-CoV)	Positive-sense ssRNA	Middle Eastern respiratory syndrome	[99]
37.	Ranunculaceae	*Nigella sativa* L. [black cumin]	Dithymoquinone	*Coronaviridae*	Respiratory syndrome coronavirus-2 (SARS-CoV-2)	Positive-sense ssRNA	COVID-19	[59]
38.	Solanaceae	*Withania somnifera* (L.) Dunal [Ashwagandha]	Withanoside V	*Coronaviridae*	Respiratory syndrome coronavirus-2 (SARS-CoV-2)	Positive-sense ssRNA	COVID-19	[64]
39.	Solanaceae	*Withania somnifera* (L.) Dunal [Ashwagandha]	Somniferine	*Coronaviridae*	Respiratory syndrome coronavirus-2 (SARS-CoV-2)	Positive-sense ssRNA	COVID-19	[64]
40.	Phyllophoraceae	*Gymnogongrusgriffithsiae* (Turner) C. Martius	Sulphated polysaccharide (named kappa carrageenan) from whole plants	*Flaviviridae*	Dengue virus type 2 (DENV-2)	Positive-sense ssRNA	Dengue (Breakbone fever)	[94]
41.	Solanaceae	*Atropa belladonna* L. [belladonna]	l-hyoscyamine, atropine, Belldonic, Scopoletin (l-methyl aesculetin), hyoscine, and pyridine and N-methyl praline	*Filoviridae*	Ebola virus	Negative-sense ssRNA	Ebola haemorrhagic fever (Ebola HF)	[100, 101]
42.	Scrophulariaceae	*Scrophularia scorodonia* L. [Balm-leaved Figwort]	Triterpene glycosides (named Saikosaponins B_2_)	*Coronaviridae*	Human coronavirus 229E (HCoV-22E9)	Positive-sense ssRNA	Nosocomial respiratory viral infection (NRVI)	[93]
43.	Chordariaceae	*Cladosiphonokamuranus*Tokida [Mozuku]	Fucoidan from whole plants	*Flaviviridae*	Dengue virus type 2 (DENV-2)	Positive-sense ssRNA	Dengue (Breakbone fever)	[94]
44.	Fabaceae	*Leucaena leucocephala* (Lam.) de Wit [white leadtree]	Galactomanan from seeds	*Flaviviridae*	Dengue virus (DENV)	Positive-sense ssRNA	Dengue (Breakbone fever)	[94]
45.	Theaceae	*Camellia sinensis* (L.) Kuntze	Epicatechin gallate	*Coronaviridae*	Respiratory syndrome coronavirus-2 (SARS-CoV-2)	Positive-sense ssRNA	COVID-19	[65]
46.	Menispermaceae	*Tinospora cordifolia* (Thunb.) Miers [Gurjo]	Tinocordiside	*Coronaviridae*	Respiratory syndrome coronavirus-2 (SARS-CoV-2)	Positive-sense ssRNA	COVID-19	[64]
47.	Fabaceae	*Mimosa scabrella* Benth. [Bracatinga]	Galactomanan from seeds	*Flaviviridae*	Dengue virus (DENV)	Positive-sense ssRNA	Dengue (Breakbone fever)	[94]
48.	Celastraceae	*Cassine xylocarpa* Vent. [Marbletree]	Pentacyclic lupane-type triterpenoids	*Retroviridae*	Human immunodeficiency virus (HIV)	Positive-sense ssRNA	AIDS	[9]
49.	Celastraceae	*Maytenus cuzcoina* Loes.	Pentacyclic lupane-type triterpenoids	*Retroviridae*	Human immunodeficiency virus (HIV)	Positive-sense ssRNA	AIDS	[9]
50.	Phyllanthaceae	*Phyllanthus emblica* L. [Indian gooseberry]	Highly oxygenated norbisabolane sesquiterpenoids (Phyllaemblicins H1-H14) from root	*Orthomyxoviridae*	Influenza A virus strain H3N2	Negative-sense ssRNA	Influenza (flu)	[9]
51.	Lamiaceae	*Ocimum sanctum* L. [Tulsi]	Vicenin	*Coronaviridae*	Respiratory syndrome coronavirus-2 (SARS-CoV-2)	Positive-sense ssRNA	COVID-19	[64]
52.	Magnoliaceae	*Magnolia officinalis* Rehder & Wilson [Houpo magnolia]	Honokiol	*Flaviviridae*	Dengue virus type 2 (DENV-2)	Positive-sense ssRNA	Dengue (Breakbone fever)	[9]
53.	Lamiaceae	*Ocimum sanctum* L. [Tulsi]	4′-O-glucoside 2^″^-O-p-hydroxybenzoagte	*Coronaviridae*	Respiratory syndrome coronavirus-2 (SARS-CoV-2)	Positive-sense ssRNA	COVID-19	[64]
54.	Fabaceae	*Tephrosia madrensis* Seem.	Methyl-hildgardtol A from leaves and flowers	*Flaviviridae*	Dengue virus (DENV)	Positive-sense ssRNA	Dengue (Breakbone fever)	[94]
55.	Lamiaceae	*Ocimum sanctum* L. [Tulsi]	Isorientin	*Coronaviridae*	Respiratory syndrome coronavirus-2 (SARS-CoV-2)	Positive-sense ssRNA	COVID-19	[64]
56.	Theaceae	*Camellia sinensis* (L.) Kuntze	Gallocatechin-3-gallate	*Coronaviridae*	Respiratory syndrome coronavirus-2 (SARS-CoV-2)	Positive-sense ssRNA	COVID-19	[65]
57.	Fabaceae	*Leucaena leucocephala* (Lam.) de Wit [White leadtree]	Epicatechin gallate	*Flaviviridae*	Yellow fever virus	Positive sense ssRNA	Yellow fever	[94]
58.	Papaveraceae	*Chelidonium majus* L. [greater celandine]	Low-sulfated poly-glycosaminoglycan moiety from freshly prepared crude extract	*Retroviridae*	Retrovirus (HIV)	ssRNA	AIDS	[27]
59.	Gentianaceae	*Swertia bimaculata* (Siebold & Zucc.) Hook. f. & Thomson ex C.B. Clarke [double-spotted Swertia]	Sesterterpenoid	*Retroviridae*	Human immunodeficiency virus (HIV)	Positive-sense ssRNA	AIDS	[27, 102]
60.	Gentianaceae	*Swertia punicea* Hemsl.	Xanthone	*Retroviridae*	Human immunodeficiency virus (HIV)	Positive-sense ssRNA	AIDS	[27]
61.	Euphorbiaceae	*Euphorbia neriifolia* L. [Indian Spurge Tree]	Diterpenoids (named eurifoloids E and F)	*Retroviridae*	Human immunodeficiency virus (HIV)	Positive-sense ssRNA	AIDS	[27]
62.	Menispermaceae	*Stephania tetrandra* S. Moore [Fen Fang Ji]	Tetrandrine, Cepharanthine, Fangchinoline	*Coronaviridae*	Human coronavirus OC43 (HCoV-OC43)	Positive-sense ssRNA	—	[70, 103]
63.	Acanthaceae	*Rhinacanthus nasutus* (L.) Kurz [Snake jasmine]	Lawsone methyl ether from leaves	*Retroviridae*	Human immunodeficiency virus (HIV)	Positive-sense ssRNA	AIDS	[27]
64.	Lauraceae	*Laurus nobilis* L. [bay tree]	Beta-ocimene, 1,8-cineole, alpha-pinene, beta-pinene in extracted oil from plant	*Coronaviridae*	Severe acute respiratory syndrome coronavirus (SARS-CoV)	Positive-sense ssRNA	Severe acute respiratory syndrome (SARS)	[51]
65.	Ranunculaceae	*Nigella sativa* L. [black cumin]	Thymoquinone, nigellimine	*Coronaviridae*	Severe acute respiratory syndrome coronavirus 2 (SARS-CoV-2),	Positive-sense ssRNA	Coronavirus disease 2019 (COVID-19)	[104]

## Data Availability

The data used to support the findings of this study are included within the article.
